# Current Advances in the Functional Genes of Edible and Medicinal Fungi: Research Techniques, Functional Analysis, and Prospects

**DOI:** 10.3390/jof10050311

**Published:** 2024-04-25

**Authors:** Wenyun Li, Gen Zou, Dapeng Bao, Yingying Wu

**Affiliations:** 1 National Engineering Research Center of Edible Fungi, Key Laboratory of Applied Mycological Resources and Utilization, Ministry of Agriculture, Institute of Edible Fungi, Shanghai Academy of Agricultural Sciences, Shanghai 201403, China; liwenyun1209@126.com (W.L.); zougen@sibs.ac.cn (G.Z.); 2 College of Food Sciences and Technology, Shanghai Ocean University, Shanghai 201306, China

**Keywords:** macrofungi, genetic transformation, gene editing, mating-type genes, active substances

## Abstract

Functional genes encode various biological functions required for the life activities of organisms. By analyzing the functional genes of edible and medicinal fungi, varieties of edible and medicinal fungi can be improved to enhance their agronomic traits, growth rates, and ability to withstand adversity, thereby increasing yield and quality and promoting industrial development. With the rapid development of functional gene research technology and the publication of many whole-genome sequences of edible and medicinal fungi, genes related to important biological traits have been mined, located, and functionally analyzed. This paper summarizes the advantages and disadvantages of different functional gene research techniques and application examples for edible and medicinal fungi; systematically reviews the research progress of functional genes of edible and medicinal fungi in biological processes such as mating type, mycelium and fruit growth and development, substrate utilization and nutrient transport, environmental response, and the synthesis and regulation of important active substances; and proposes future research directions for functional gene research for edible and medicinal fungi. The overall aim of this study was to provide a valuable reference for further promoting the molecular breeding of edible and medicinal fungi with high yield and quality and to promote the wide application of edible and medicinal fungi products in food, medicine, and industry.

## 1. Introduction

The term “edible and medicinal fungi” broadly refers to macrofungi that are highly beneficial for human consumption or medicinal purposes and are characterized by their nutrient richness, delightful flavor, and a range of substances that are advantageous for human health. In the dry matter of edible and medicinal fungi, proteins account for 15–35%, carbohydrates account for 51.3–62.5%, and dietary fibers account for 8–10.4% [1,2], with a sufficient content of essential amino acids to complement vegetables, legumes, and dairy products [3]. Edible and medicinal fungi contain polysaccharides, antifungal proteins [4], terpenoids, steroids, adenosine, and other bioactive substances, offering antioxidant, anti-aging, anti-tumor, and antivirus benefits and reducing blood lipids, lowering blood pressure and cholesterol, liver-protective detoxification, immune control, and other vital roles [5,6,7,8]. Almost 150,000 species of fungi have been described, of which more than 2000 are edible species prevalent in Asia, Europe, America, Africa, and Oceania [9]. Currently, more than 100 types of edible and medicinal fungi have been cultivated artificially, among which *Lentinula edodes*, *Pleurotus* spp., *Auricularia* spp., *Agaricus bisporus*, and *Flammulina filiformis* are the main cultivars in the world [10]. In addition to their food and medicinal value, edible and medicinal fungi can utilize agricultural and forestry wastes as a source of cultivation substrates, compost, feed, or biofuel [11], playing an important role as a core link in the circular agricultural economy [12], can simultaneously realize the ultimate goals of economic development and environmental protection [13].

Despite the rapid development of the edible and medicinal fungi industry worldwide, problems, such as a single genetic background of factory-produced varieties, a high degree of product homogenization, and easy degradation of strains, have seriously constrained the sustainable and healthy development of the industry. Currently, the cultivation of high-yield and high-quality edible and medicinal fungi varieties and the development of high-value products are the most economical and effective ways to solve the problems of the edible and medicinal fungi industry. Basic scientific research on edible and medicinal fungi is closely related to their industrial development, and these industrial issues provide important directions for scientific research on edible and medicinal fungi. The growth cycle, agronomic traits, intrinsic quality, and other important traits related to the edible and medicinal fungi industry are subject to complex genetic regulation, and the screening and identification of relevant functional genes is a key step in the study of the molecular mechanisms of the biological phenogenesis of various edible and medicinal fungi and is one of the important aspects of the construction of the discipline of edible and medicinal fungi genetics [14]. However, constrained by complex genetic backgrounds and small research populations, the established technological system for conventional functional gene research in plants and other microorganisms cannot be directly applied to most edible and medicinal fungi species, particularly Basidiomycota. In recent years, with the rapid development of molecular sequencing technology and the successful application of modern biological techniques, such as gene editing, important functional genes in edible and medicinal fungi, including genes related to the development of mycelia and fruiting bodies, those regulating the synthesis of active substances, those for mating type, and those for environmental response have been analyzed step-by-step. With an increasing number of research teams focusing on the research field of edible and medicinal fungi functional genes and achieving many scientific advances, a body of work has been gradually formed based on edible and medicinal fungi germplasm resources, multi-omics data, and the evaluation of important agronomic traits. 

**Figure 1:**
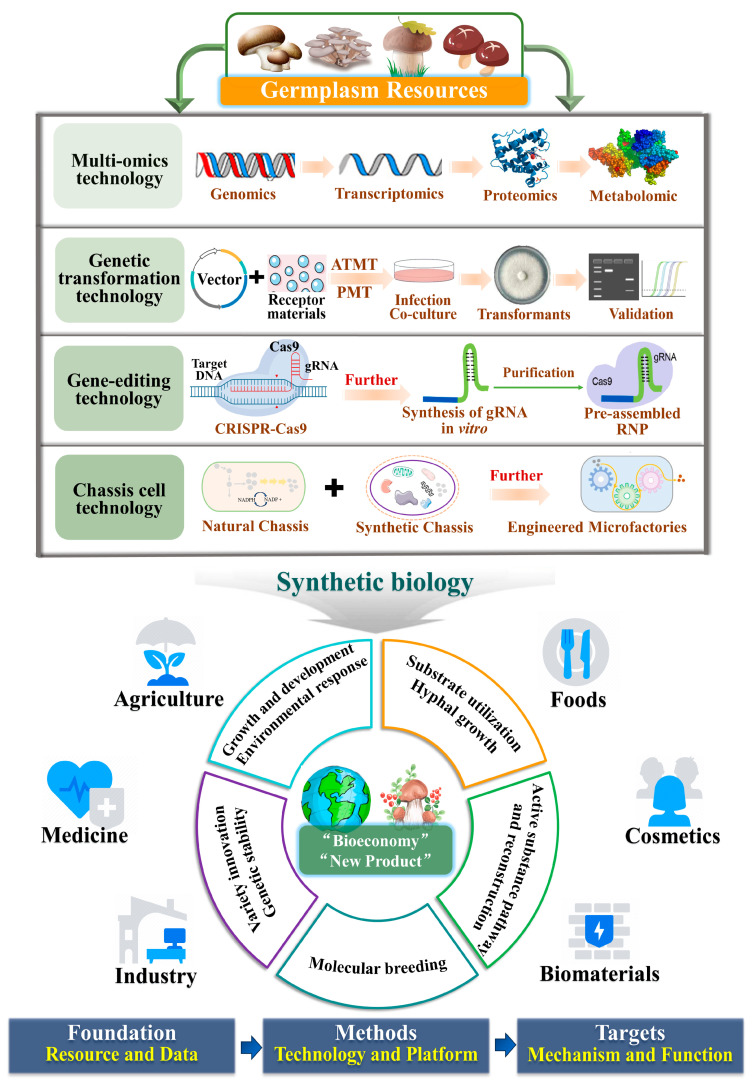
The ideas and applications of functional gene analysis technology in edible and medicinal fungi research.

Using the technologies of multi-omics, genetic transformation, gene-editing, chassis cell as a platform, and adopting synthetic biology strategies through the development and application of important functional genes will ultimately realize a circular economy and a variety of innovations (Figure 1).

This paper summarizes and compares the main techniques applied by global researchers in recent years in the field of functional gene research in edible and medicinal fungi and analyzes the important identified gene functions to lay a scientific foundation for further elucidation of the intrinsic mechanism of the life activities of edible and medicinal fungi. This paper aims to provide a theoretical reference for future germplasm innovation and cross-disciplinary application of edible and medicinal fungi products.

## 2. Advances in Functional Gene Research Techniques in Edible and Medicinal Fungi

The screening and characterization of functional genes are the basis for studying how genes regulate important phenotypes in edible and medicinal fungi. The successful completion of this work relies heavily on the development and application of relevant bioinformatics and biological experimental techniques. In edible and medicinal fungi research, to isolate a certain functional gene for a certain purpose, we usually start from a specific phenotypic trait and use omics technology methods, such as genetic map construction, genome-wide association analysis, and expression profiling difference analysis, to mine and screen the associated genes and further use genetic transformation, gene editing, and chassis cell series technology to functionally resolve and validate the candidate genes.

### 2.1. Multi-Omics Technology

Evolving omics technologies are effective aids in resolving important phenotype-associated functional genes in edible and medicinal fungi, including genomics, pan-genomics, transcriptomics, proteomics, and metabolomics. Zhao et al. [15] sequenced and annotated the whole genome of *Morchella esculenta* MT1, revealing that its functional genes were focused on metabolic processes, cellular processes, and binding and catalytic activity processes, and it was hypothesized that this mushroom has stronger low-temperature and cold-resistant properties. With the development of genomics, the construction of pan-genomic data accelerated the resolution of the genetic mechanisms underlying the formation of agronomic traits in edible and medicinal fungi. Drott et al. [16] studied the pan-genomes of the macrofungi *Amanita phalloides* and *Agaricales* and reported that the differential expression of MSDIN, a gene encoding a toxic secondary metabolite, leads to different phenotypes. They further revealed the dynamics of the toxin gene in natural evolution, providing an effective pathway for exploring secondary metabolites in other Basidiomycota. The pangenome of *Auricularia heimuer* constructed by Guo et al. [17] included 14,089 gene families, 67.56% of which were core gene families and 31.88% of which were minor gene families. The genome was screened for the chitinase gene *ahchi1* and a carbohydrate-binding module (CBM)-related gene, which provided a reference for genes related to substrate utilization in edible and medicinal fungi.

Transcriptomics focuses on the overall study of gene transcription and regulation in cells, thereby revealing the molecular mechanisms of a particular biological process. Light plays a specific role in the regulation of secondary metabolites in edible and medicinal fungi, but the molecular mechanisms and light-induced genes are still unclear. Kim et al. [18] compared the transcriptome data of mushrooms under blue light and darkness; screened 221 upregulated genes and 541 downregulated genes; and reported that the DDR48-heat-shock proteins, 12 kD heat-shock proteins, and conserved fungal proteins were highly expressed under blue-light irradiation. This provides important information for the understanding of blue-light sensing and receptor mechanisms in fungi. Hao et al. [19] analyzed the transcriptome at three developmental stages—the mycelium, primordium, and fruiting body stages—and identified 9690 differentially differentiated genes mainly involved in oxidoreductase, biodegradation, energy metabolism, and hydrolase activity, among which CO-sensing-related genes (CA-2, CA-3, PKA-1, and PKA-20), heat-shock protein genes (HSP60 and HSP90), and transcription factors (steA, MYB, nosA, HAP1, and GATA-4/5/6) play key roles in protoplast formation, supporting the further study of substrate development. Shen et al. [20] sequenced the transcriptomes of *L. edodes* substrates (cap and stipe) at different developmental stages and showed that genes encoding carbonic anhydrase, chitinase, GH5, and cytochrome P450s may function in stipe elongation.

Proteomics is a pathway for the comprehensive understanding of molecular mechanisms, and this type of research brings “life” to genomics [21]. To date, more than 800 edible and medicinal fungi have been reported to possess important pharmacological properties, such as anti-tumor and immunomodulatory activities [22]. The use of proteomics technology allows the identification of a large number of differentially regulated proteins, providing an important platform for the discovery of potential therapeutic targets. Lin et al. [23] determined changes in the proteomic profiles of LLC1 cells carrying tumor lesions in mice treated with *Ganoderma lucidum* fungal immunomodulatory proteins (LZ-8) and reported that HSP60 and HSP70 inhibitors effectively inhibited cell migration and reduced cell viability, suggesting that HSPs may be associated with anti-lung cancer activity. To gain insight into the mechanism of action of different light sources on the growth and development of edible and medicinal fungi, Zhu et al. [24] compared the protein-expression profiles of comparative proteomic analysis, revealing differential protein expression of *Hypsizigus marmoreus* in response to different light qualities under different colors of light (white, red, green, blue, and black) based on proteomics technology; KEGG enrichment was shown to be related to the pathways of MAPK, light stress, proteasome, glycolysis, and carbohydrate-active enzymes. *Coprinus comatus* is a perishable and difficult-to-preserve edible and medicinal fungi owing to its high moisture content and high respiration rate. Qu et al. [25] used a tandem mass tags-based quantitative proteomic technique to analyze the proteomic response of post-harvested *C. comatus* stored for 16 d at 8 ± 1 °C, and the results showed that differential abundance proteins (DAPs) were mainly involved in ribosome, citrate cycle, glycolysis, and glutamate metabolism.

The secondary metabolites of edible and medicinal fungi have important applications as food additives, pharmaceutical intermediates, bio-based materials, and dietary amino acids [26]. Metabolomics technology can effectively analyze the metabolite species, content, and differences in edible and medicinal fungi. Jiang et al. [27] analyzed the metabolomics of *G. lucidum* after treatment with methyl jasmonate (MeJA) for 24 h using gas chromatography–mass spectrometry (Agilent, Palo Alto, CA, USA) and liquid chromatography–mass spectrometry (Thermo Fisher Scientific, Waltham, MA, USA) and reported that its cellular energy metabolism shifted from basal metabolism to secondary metabolism, confirming that MeJA was involved in the promotion of ganoderic acid synthesis. *G. lucidum* extract can be used as a fermentation medium to promote the growth of probiotics. Liu et al. [28] analyzed the effect of *G. lucidum* aqueous extract (GLE) on key metabolites of *Lactobacillus rhamnosus* GG (LGG) based on non-targeted metabolomics and identified different differential metabolites, such as pyrotechnic acid, taurocholic acid, and estradiol, which are mainly involved in metabolic pathways such as tyrosine metabolism, pyrimidine metabolism, and alanine and aspartate enrichment, providing theoretical support for using GLE to improve probiotic production. Zeng et al. [29] analyzed the metabolite changes of *Dictyophora indusiate* under three drying methods (vacuum freeze-drying FD, vacuum drying VD, and hot-air drying HD) based on quantitative metabolomics. They reported that acidity was provided by organic acids produced in the tricarboxylic acid cycle, and browning was caused by the melted reaction, the oxidative degradation of ascorbic acid, and the metabolic pathways mainly catalyzed by endogenous enzymes mainly in the phenylalanine metabolic pathway.

The results of the multi-omics joint analysis can be corroborated with each other, which can help improve the scientificity and credibility of the study and will be more widely and deeply applied to the analysis of edible and medicinal fungi functional genes in the future. Sun et al. [30] utilized the genome and transcriptome to gain insight into the molecular mechanism of nucleus formation in *Pleurotus tuber-regium* and reported that the genome size was 35.82 Mb and encoded 12,173 putative proteins. Of these, 1146 and 1249 genes were upregulated and downregulated, respectively, and it was hypothesized that these differential genes were related to substrate catabolism, oxidation–reduction process, cell wall synthesis, and other biological processes. Cai et al. [31] combined metabolomics and transcriptomics technology to study the browning of *A. bisporus*, which revealed that the genes *AbPPO2*, *AbPPO3*, and *AbPPO4* were involved in the browning-related pathway and the resistance to browning could be judged by the expression level of *AbPPO*. Furthermore, metabolomics revealed that the metabolites of organic acids in vivo further inhibited the activity of PPO by lowering the pH value. The results of this study are significant for the adoption of appropriate measures to mitigate mushroom browning. Functional-omics technologies are the key to link gene expression and phenotype. Marian et al. [32] performed comparative genomics and transcriptomics analysis of two wild strains of *Schizophyllum commune* and identified *roc1* as a key regulator of cellulose degradation in basidiomycetes for the first time. This model species can provide insights into cellulose degradation in basidiomycetes. In addition, advanced functional-omics technologies can be used to analyze light-responsive regulatory genes. To provide a reference for light conditions in the process of factory cultivation, Liu et al. [33] used gene expression profiling and Chip-Seq to study the molecular mechanism of GATA transcription factor *CcNsdD2* from *Coprinopsis cinerea* in hypha kinking and light morphogenesis. The phenomenon of low-temperature autolysis in *Volvariella volvacea* seriously hinders the postharvest storage and marketing of commercial edible and medicinal fungi; therefore, our team analyzed the gene and metabolite expression profiles of *V. volvacea* after treatment with the L345-0044 inhibitor at 4 °C using absolute quantitative transcriptome and metabolome analysis [34]. The two key enzymes, tyrosinase and β-glucosidase, were screened out. The inhibitor treatment revealed that the activities of these enzymes were reduced by 27.25% and 73.20%, respectively, which is favorable for slowing down low-temperature autolysis.

In summary, whole-genome sequences can be used to gain insight into genetic data, transcriptomics can be used to analyze differentially expressed genes in different phenotypes of the same edible and medicinal fungi, and proteomics can be used to explore the biosynthetic pathways of amino acids and enzymes. Metabolomics technology can be used to predict substance metabolic pathways and identify metabolomic markers in large edible and medicinal fungi. Despite the many benefits, there are still certain limitations to genomics technologies. For example, genomics can only provide information about metabolic potential, metabolomics and proteomics show poor reproducibility, there are limitations in multi-omics technology in annotating and interpreting the functions of individual genes and molecules, and omics technologies are relatively homogenously applied [35]. Most of the genomes of edible and medicinal fungi that have been sequenced thus far are single genomes, which do not cover a wide range of genetic diversity and are difficult to study. In the future, pan-genomic studies of edible and medicinal fungi should be strengthened and combined with the joint application of multi-omics technology to facilitate a more comprehensive and precise identification of genes involved in important biological processes at the bioinformatics level. Additionally, there is a need to focus on combining wet and dry experiments to provide more reliable evidence of gene function supported by molecular genetic techniques.

### 2.2. Molecular Genetic Techniques in Edible and Medicinal Fungi

#### 2.2.1. Genetic Transformation Technology

Genetic transformation technology for edible and medicinal fungi is used to obtain strains with targeted changes in genetic traits by integrating target fragments into vectors using molecular biology techniques and genetic engineering, followed by transformation into recipient cells [36]. Compared to unicellular microorganisms, such as *Escherichia coli*, edible and medicinal fungi have a complex genetic background, specific morphology, and thicker cell walls, which makes it more difficult to introduce exogenous gene fragments. Therefore, the development of efficient genetic transformation techniques is an effective way to explore the functional genes in edible and medicinal fungi. Genetic transformation techniques for edible and medicinal fungi include *Agrobacterium tumefaciens*-mediated transformation (ATMT), protoplast-mediated transformation (PMT), liposome-mediated transformation (LMT), and electroporation transformation (EP) [37]. The advantages, disadvantages, and applications of these approaches are summarized in Table 1. ATMT and PMT are more commonly used in laboratories because they combine conversion efficiency, stability, ease of operation, and equipment requirements.

ATMT technology has been used in combination with target gene overexpression or RNA interference methods to study gene functions related to growth and development, environmental stress responses, and the metabolism of functional, active ingredients in a variety of edible and medicinal fungi (Table 2). Overexpression involves placing the target gene downstream of a strong promoter or an inducible promoter to enhance the expression of the target gene, whereas RNA interference technology silences the target gene by constructing a hairpin or reverse double promoter structure. As can be seen from Table 2, many studies have been conducted on *F. filiformis*, *L. edodes*, and *G. lucidum*. For example, *fvhom1*, *Ste12-like*, *FIP-fve*, and *FfGal* in *F. filiformis* were overexpressed by ATMT, and RNA interference was performed on *FIP*, *FvHmg1*, *Fv-ada*, and *FfGal*. These genes are involved in the growth, development, and environmental stress of *F. filiformis*. *Hsp20* and *LeHD1* were overexpressed in *L. edodes*, and RNA interference was performed on *LeHD1*, *LELCRP1*, *LeDnaJ*, *TrpE*, *YUCCA*, and *LetrpB*. These genes are involved in the growth, development, biodegradation, and environmental stress responses of *L. edodes*. Based on the overexpression of *D9desA*, *Gl-aact*, and *HMGR* in *G. lucidum*, these genes participate in the biosynthesis of functionally active substances.

Conversion efficiency is an important index for measuring ATMT technology, and studies have shown that the promoter, receptor material, and *Agrobacterium tumefaciens* type are important influencing factors [64]. Promoters are important cis-acting elements that control gene transcription, and there may be significant differences in the levels of exogenous gene expression driven by different promoters. Lv et al. [65] utilized a binary vector consisting of polyubiquitin and the GPD promoter in conjunction with the ATMT method to successfully achieve the genetic transformation of *M. esculenta*. Our team constructed two plasmids with *CaMV35s* and *H. marmoreus* GPD as promoters and reported that the latter increased the expression levels of exogenous genes by 22–36 times [66]. The genome of *Pleurotus eryngii* contains two *gpd* genes, of which *gpd1* is significantly more highly expressed than *gpd2* at different developmental stages and drives the expression of exogenous genes more efficiently [67]. Receptor materials commonly used for ATMT in edible and medicinal fungi include mycelia, mononucleate spores, and protoplasts, with varying conversion efficiencies. Liu et al. [68] used the mycelia of *A. bisporus* as the receptor material and a colonized millet grain method for transformation by *Agrobacterium*, which resulted in a transformation efficiency of up to 53.85% with more than 85% of the transformants remaining mitotically stable for five consecutive rounds of culture. Liu et al. [69] used the mycelial fragments and protoplasts of mononuclear dans and binucleate G1 as conversion receptors and reported that the mycelial fragments of the binucleate had the highest conversion efficiency, up to 40.31%.

Differences in the efficiency of genetic transformation mediated by *A. tumefaciens* were observed. In *P. ostreatus*, the conversion efficiencies from highest to lowest are *LBA4404*, *GV3101*, *EHA101*, and *EHA105* [70]; therefore, selecting suitable strains is conducive to genetic transformation. In addition, the mycelium medium and receptor genotype play key roles in conversion efficiency. Yan et al. [71] established a genetic transformation system for the mononuclear and binuclear bodies of *L. edodes* and reported that the conversion efficiency of the grain medium was the highest, up to 75.48%, and the conversion efficiency of binuclear bodies was higher than that of mononuclear bodies, providing a new method for the genetic transformation of edible and medicinal fungi. Since the first successful transformation in yeast in 1978, the PMT method has gradually become a common cell fusion technology based on the principle that the formation of precipitates on the cell surface by PEG and divalent cations, such as Ca^2+^, Mn^2+^, and Mg^2+^, with exogenous DNA alters the permeability of the membrane and facilitates the entry of exogenous DNA. The green fluorescent protein reporter gene was transferred into *Agrocybe aegerita* using the PMT method. The results showed that the expression of the *GFP* gene was independent of introns, laying a foundation for subsequent research on functional genes [72]. Antroquinonol (AQ) is an important biologically active constituent of *Antrodia cinnamomea* with antiviral, nephroprotective, and neuroprotective effects [73]. Liu et al. [74] transferred and overexpressed *ubiA* and *CoQ2* genes, which enhance antroquinonol production via the overexpression of 4-hydroxybenzoate polyprenyltransferase biosynthesis-related genes in *A. cinnamomea*; the concentration of AQ was 2- and 2.61-times higher than that of the control after fermentation, respectively. *Pleurotus ferulae*, a unique edible fungus from Xinjiang, China, can effectively degrade lignin. However, its low conversion efficiency has limited the systematic study of its molecular mechanisms and metabolic control. Therefore, Zhang et al. [75] established the PMT conversion method in *P. ferulae* for the first time and reported that adding 50 μg SS-DNA, 70 μg λDNA, and 0.4 μmol spermidine could maximize the conversion efficiency. An overexpression system containing three different endogenous promoters (P*_PFGPD_*_1_, P*_PFGPD_*_2_, and P*_PFSAR_*_1_) was established to drive the expression of different genes, promoting the expression of functional genes and novel secondary metabolic pathways.

Currently, the main screening markers used in ATMT technology are antibiotics, metabolites, nutritional deficiencies, herbicides, and fungicide-resistance genes, which may pose threats to environmental and food safety. Therefore, the selection of biosafety genes as screening markers is a focus for the optimization of edible and medicinal fungi genetic transformation technologies in the future. Our team used *Pesdi1*, an autosomal mutated gene in *P. eryngii*, as a safety-selective marker and achieved ATMT in *P. eryngii*, which provided a reference for the development of biosafety markers in other edible and medicinal fungi [76]. However, the molecular mechanism of T-DNA transfer and integration in fungal receptors is different from that of plants, which needs to be further explored to determine its signaling and interaction patterns and to improve the expression of exogenous genes in edible and medicinal fungi [77]. In addition, the key to genetic transformation is the preparation and regeneration of protoplasts; the age of the bacteria, enzyme system, osmotic stabilizers, enzyme digestion time, and temperature will affect the quantity and quality of protoplasts [78], and conditions should be optimized and explored for different edible and medicinal fungi for genetic transformation to achieve the best efficiency.

#### 2.2.2. Gene-Editing Technology

Gene-editing technology uses artificially designed or modified nucleases to make precisely targeted modifications to the genome. Double-stranded DNA repair is performed by cutting a specific site using homology-directed repair (HDR) or non-homologous end joining (NHEJ) [79]. CRISPR/Cas9, a third-generation gene-editing technology with the advantages of simple operation, high precision, and low cost, has been widely used for gene editing in edible and medicinal fungi in the past few years, such as in *A. bisporus*, *G. lucidum*, *C. cinerea*, and *C. militaris*.

*A. bisporus* was the first edible fungus to undergo gene editing using the CRISPR/Cas9 system. In 2016, Yang et al. [80] used this system to knock out one of six genes encoding polyphenol oxidase (PPO) in *A. bisporus*, thereby reducing PPO enzyme activity by 30%, effectively slowing down postharvest browning and extending shelf life. The announcement by the United States Department of Agriculture (USDA) in the same period confirmed the feasibility of gene editing in crop applications, and since then, it has been progressing toward an era of sophisticated molecular breeding, gradually paving the way for the era of synthetic biology.

*G. lucidum* is an important medicinal fungus, and the development of a suitable gene-editing system can provide an efficient and feasible pathway for the efficient synthesis of secondary metabolites. Professor Zhong Jianjiang’s group at Shanghai Jiaotong University successfully established a CRISPR/Cas9-mediated gene-editing system in *G. lucidum* for the first time [81], successfully controlled the expression of Cas9 using the *G. lucidum gpd* promoter and *Trichoderma reesei pdc* terminator, adopted the T7 promoter for in vitro transcription of sgRNA, successfully transcribed them into Cas9 protein-expressing strains, and ultimately succeeded in obtaining uracil nutritionally deficient strains. Wang et al. [79] studied *cyp515018*, a gene encoding cytochrome P450 monooxygenase, using the CRISPR/Cas9 system and reported that the production of ganoderic acid was significantly reduced in the mutant compared to the wild type. Liu et al. [82] added introns upstream of the *Cas9* gene to reach 14–18 *ura3* mutants, whereas the use of double sgRNA in *G. lucidum* resulted in the deletion of *ura3* and *GL17624* gene fragments, effectively expanding the library of mutants in *G. lucidum*. Using the PMT of *G. lucidum*, Zhang et al. [83] obtained a strain containing a complete *cas9* expression box and optimized this using 0.006% Tratone X-100, increasing the number of *ura3* mutants from 4 to 18 and providing a more efficient method for future studies of gene function and molecular breeding.

*C. cinerea* is a model organism that can help researchers understand the growth, development, and reproductive mechanisms of other edible and medicinal fungi. Sugano et al. [84] successfully applied CRISPR/Cas9 gene-editing technology in this species using the *CcDED1* promoter to express codon-optimized *Cas9* genes from humans, Candida, Arabidopsis, and Basidiomycota and ligated the same vector with sgRNA expression elements. Only 19 transformants from *C. cinerea* were observed to have reduced green fluorescent protein. The attenuation of green fluorescent proteins was detected in the two transformed strains, with an editing efficiency of 10.5%. Yu et al. [85] transformed in vitro-expressed Cas9 and sgRNAs into *C. cinerea* via protoplasts and did not detect *pyrG* editing, which was hypothesized to be due to the degradation of Cas9 proteins and sgRNAs during the transformation process or because the buffer used in vitro cleavage experiments was unfavorable for the assembly and zymography activities.

*C. militaris* is an edible medicinal fungus, and the successful application of gene-editing technology has accelerated the development and utilization of its active substances. Chen et al. [86] used PMT to introduce fragments containing *Cas9* and *GFP* fusion genes into Cas9 protein-expressing strains and successfully mutated the target site with an editing efficiency of 11%. In the same year, Meng et al. [87] constructed an efficient gene-editing system based on autonomously replicating plasmid with an AMA1 sequence for the first time in *C. militaris* and, through homologous directed repair, the efficiency of *Cmwc-55* and *Cmvvd* reached 1.89% and 1%, respectively. The simultaneous editing efficiency of dual genes was 10%. This technique can be used for the simultaneous modification of multiple genes in functional gene research.

Subsequently, researchers developed a pre-assembled Cas9-sgRNA ribonucleoprotein (RNP) technology based on the CRISPR/Cas9 gene-editing technology. These two principles are similar, but RNPs can be incubated with purified Cas9 protein and synthesized sgRNA in vitro to form the RNP complex without plasmid construction during the experiment, which brings the technical advantages of simplicity and high universality [88]. In 2017, Al Abdallah et al. [89] successfully edited *pksp*, a gene related to melanin synthesis in *Aspergillus fumigatus*, using an RNP system. In 2019, Jan et al. [90] constructed an RNP system for *S. commune*, a model organism of edible and medicinal fungi, and successfully edited the effective editing of transcription factor *hom2*. In 2021, our team optimized the RNP system by adding the surfactant Triton X-100, which increases the proportion of homonuclear transformants in filamentous fungi [91]. Subsequently, in cooperation with other teams, the RNP complex-mediated HR system effectively knocked out three target genes of alkaloid synthesis in *Claviceps purpurea*, with editing efficiencies ranging from 50% to 100%, which greatly facilitated the study of the ergot alkaloid synthesis pathway [92]. In 2022, Liu et al. [93] optimized a CRISPR/Cas9 gene-editing method for the in vitro assembly of ribonucleoprotein complexes in *F. filiformis* with the addition of Triton X-100. This resulted in editing efficiencies of up to 5% and the more rapid growth of transformants compared to common gene-editing techniques. In 2023, Boontawon et al. [94] established a system of RNPs in *P. ostreatus* without exogenous DNA genes, and double-gene-edited mutants were obtained by screening RNP-added strains for 9-FOX resistance.

The genetic transformation of edible and medicinal fungi is relatively difficult, and with the advent of the post-genomic era, many predicted and annotated functional genes have been successfully validated using gene-editing techniques. However, the CRISPR/Cas9 system faces several challenges, including low editing efficiency, poor accuracy, off-target effects, and difficulties in screening mutants [95]. In the future, we can improve the editing efficiency by optimizing the codons of target species and selecting appropriate promoters to express *Cas9* genes; improve the accuracy by inhibiting NHEJ repair or enhancing HDR repair; detect off-target effects on time using chromatin immunoprecipitation sequencing (ChIP-seq) and genome-wide, unbiased identification of DSBs enabled by sequencing (GUIDE-seq) methods, optimized sgRNA sequences, and the modification of Cas9 proteins to reduce off-target effects; and the addition of chemical reagents to increase the proportion of mononuclear protoplasts to improve the efficiency of obtaining pure transformants, thus reducing the difficulty of screening [96].

### 2.3. Chassis Cell Technology

Chassis cells are the host cells where metabolic reactions occur and are the reaction platforms into which systems of natural or synthetic functionalized components, circuits, and pathways are placed for rational design purposes [97]. Introducing target genes/gene clusters obtained from screening or localization into adapted chassis cells to achieve heterologous expression is an important method of verifying gene functions [98]. To date, chassis cell systems used in functional gene studies of edible and medicinal fungi include *E. coli*, *Saccharomyces cerevisiae*, *Pichia pastoris*, and *T. reesei*.

For a long time, *E. coli* has been used as a model strain for genetic and metabolic engineering because of its simple culture conditions, short growth cycle, and clear genetic background, making it an ideal host for heterologous expression. Adams et al. [99] cloned psilocybin synthesis genes (*psiD*, *psiK*, and *psiM*) and heterologously expressed them in *E. coli*, which increased the yield of psilocybin to 1.16 g/L at the fermenter level and improved the feasibility of industrial production. To address the problem of unclear key enzymes in the sugar donor synthesis pathway of *G. lucidum* polysaccharides, Li et al. [100] cloned three key enzyme genes of *G. lucidum* polysaccharides (*gl-pgm*, *gl-ugpg*, and *gl-pmi*) and induced expression and mass preparation in *E. coli* with the specific enzyme activities of 4.75, 6.26, and 13.68 U/mg after purification, which further provides theoretical bases for the development of efficient fermentation regulation strategies. Fungal glucans play key roles in providing energy and maintaining cellular structures; however, their biosynthetic mechanisms need to be elucidated. Liang et al. [101] optimized the (UDP)-glycosyltransferase *GFUGT88A1* in *Griflola frondosa* and successfully heterologously expressed it in *E. coli*. The molecular weight of the purified protein was 51.7 kDa, which provides a reference for the resolution of the glucan biosynthesis pathway in edible and medicinal fungi.

Yeast and edible and medicinal fungi, both eukaryotes, are widely used because their secretory pathways are capable of protein processing and post-translational modification without endotoxins. Wang et al. [102] screened cytochrome P450 monooxygenase (CYP450) candidate genes in *G. lucidum* using *S. cerevisiae* as a host and reported, for the first time, that one of them, *cyp5150l8*, could produce anti-tumor ganoderic acid in this host at a concentration of 5.120 mg/L after 5 days of liquid fermentation. This provides an effective strategy for optimizing heterologous cell factories for ganoderic acid production. Bai et al. [103] cloned *CDA3*, the coding gene for a novel chitin deacetylase in *C. cinerea*, and introduced it into *P. pastoris* GS3 for successful expression. The affinity chromatography-purified CDA3 protein yielded 0.067 mg/mL, with a molecular weight of approximately 27 kDa, which has the potential to be further applied to the large-scale production of chitosan. Yang et al. [104] cloned the glutamine transaminase (TGase)-coding gene *tgM* from *C. militaris* and expressed it in *P. pastoris* GS115, with an enzyme activity of 100 U/L in the supernatant of recombinant yeast fermentation. This provides a reference for the heterologous expression of TGase and its potential industrial application. Lee et al. [105] cloned *cml*, the lipase-coding gene of *C. militaris*, and introduced it into *P. pastoris* X-33 for heterologous expression and reported that the specific activity of the purified protein was 6.52 ± 0.26 U/mg, which showed a stronger catalytic efficiency compared with that of the unpurified protein and is expected to be developed as a biocatalyst.

*T. reesei* is the main industrial production strain of cellulase and hemicellulose, with strong protein-secretion ability, and is one of the most widely researched industrial fungi, with more than 80 years of research and application development. Dong et al. [106] expressed the optimized laccase-encoding gene *pox1* from *P. ostreatus* in *T. reesei* and reported that the exogenous laccase gene could be efficiently secreted and expressed with an enzyme activity as high as 237.1 IU/mL. Zhou et al. established a gene-editing system in *T. reesei* via the in vitro transcription of codon-optimized Cas9 and gRNA, and multiple genome edits with three targets were obtained based on PMT [107].

Chassis cell technology has many benefits, but there are still challenges in the heterologous synthesis of a few active secondary metabolites, such as alkaloids and phenols, including a lack of precise regulation, complex gene structure, and antibacterial properties of bioactive natural products that are toxic to chassis cells [12]. Therefore, the development of chassis cells with better compatibility for edible fungal metabolites can better realize the large-scale production of complex active metabolites from edible and medicinal fungi using synthetic biology technology.

### 2.4. Synthetic Biology

Synthetic biology is a discipline that combines observational and analytical methods in life sciences with design thinking in engineering to create new biological components, devices, and systems or redesign and adapt existing living systems in nature. Compared to other microorganisms, edible and medicinal fungi have large genomes and complex metabolic regulatory networks, and high-throughput screening of biosynthetic gene clusters to promote the production of active substances is extremely important.

The use of edible and medicinal fungi, such as *G. lucidum*, *C. militaris*, and *H. erinaceus*, can synthesize a variety of drugs and develop high-value-added cell factories based on the active substances of edible and medicinal fungi. Ganoderic acid is a series of highly oxidized secondary metabolites naturally produced by the edible and medicinal fungi *Ganoderma*. It is difficult to elucidate the biosynthetic process of ganoderic acid by analyzing the function of the *P450* gene, which is responsible for the multi-step oxidation. Zhong Jianjiang’s group at Shanghai Jiao Tong University and the Tianjin Institute of Industrial Biotechnology cooperated in establishing a high-throughput automated screening platform [108], which utilized a tunable expression strategy, functionally characterized hundreds of cytochromes *P450* from *G. lucidum* in *S. cerevisiae*, excavated and discovered key genes of the type-II ganoderic acid biosynthesis pathway, and efficiently biosynthesized the genes in heterologous form in *S. cerevisiae*. The highest content of ganoderic acid reached 56.44 mg/L after liquid fermentation for 144 h [109]. This work shows the potential and application value of synthetic biology in the discovery of novel natural products. Chen et al. [110] developed a marker-free CRISPR-Cas9-TRAMA genome-editing system for *C. militaris* to achieve the precise editing of multiple genes and the deletion of large clusters of genes, which can be used for mining and utilizing medicinal metabolic pathways. *Aspergillus oryzae* has a complete translational modification system; strong protein production and secretion capacity; secretes amylase, protease, pectinase, and saccharification enzymes; and is widely used in cell factories to produce a variety of enzyme preparations. Han et al. [111] identified a gene, *herA*, encoding orsellinic acid (OA) polyketide synthase in *H. erinaceus* and successfully heterologously expressed it in *A. oryzae*; the rate of OA production reached 57.68 mg/L by liquid fermentation when maltose was used as the carbon source, which could provide a valuable reference for the biosynthesis of important pharmaceutical intermediates.

In addition to promoting the precise breeding of edible and medicinal fungi and optimizing their growth, synthetic biology strategies can be used to mine a variety of biological components in large fungi, including edible species. Based on microbial chassis cells with excellent performance, an efficient green biofuturing technology has been developed to synthesize high-value natural drug-active molecules, which has gradually become a research hotspot in the field. Several studies have shown that biosynthetic gene clusters can be randomly activated by modifying the expression of signaling pathways, cell wall structures, and stress response elements using synthetic biology strategies. In the future, based on a deeper understanding of the gene-splicing mechanism, we can study and develop synthetic components that can be effectively used in multi-gene expression systems, construct efficient chassis that can be easily modified and screened in high throughput [112], and realize the synthesis of large quantities of target active molecules and their derivatives from edible fungus sources. The efficient degradation of SMS could be realized to significantly contribute to the healthy development of the edible fungus industry under the goal of “carbon neutrality” and the creation of a high-economic-value industry.

## 3. Advances in the Analysis of Important Functional Genes of Edible and Medicinal Fungi

Through the continuous development and optimization of various functional gene research techniques in edible and medicinal fungi, the functions of a few important genes have been elucidated, including genes related to mating type, mycelial growth, substrate utilization, nutrient transport, environmental response, and the synthesis and regulation of important active substances. The expression of these genes directly or indirectly affects important traits, such as the growth cycle, yield, quality, and active ingredients of edible and medicinal fungi. Compared with the traditional methods of edible and medicinal fungi breeding, the exploration and analysis of functional genes will provide new ideas for modernized molecular and design breeding.

### 3.1. Mating-Type Genes

In edible and medicinal fungi, mating-type genes control the mating behavior between amenable monokaryosomes and the expression of downstream genes that ultimately lead to the production of sexual spores. The mating-type genes of edible and medicinal fungi such as *C. militaris*, *M. esculenta*, *F. filiformis*, *L. edodes*, and *P. eryngii* are now known.

The sexual reproduction of Ascomycota is controlled by mating-type loci that contain two allogeneic genes—*MAT1-1* and *MAT1-2*. The degradation of *C. militaris* strains cannot be ignored and can lead to a slow growth rate, low conidial production, reduced number of primordia, a prolonged growth cycle, and reduced secondary metabolism, resulting in significant economic losses [63]. Feng et al. [113] pre-screened the excellent traits of artificially cultivated *C. militaris* and isolated two different mating-type genes, *Mat-1* and *Mat1-2*, from 72 monospore strains. They conducted cultivation experiments to show that only strains with different mating-type genes can develop into fruition bodies, which provides a theoretical basis for preventing the degradation of *C. militaris* during large-scale cultivation. To increase the chance of success of single-spore cross-breeding of *M. esculenta*, Guo et al. [114] screened *MAT1-1* and *MAT1-2* mating types, a total of 20 single mating-type hybrid parents with good culture traits, and hybrid strains of mating-type genes. They reported that the fungus cap is large, and the stipe is small, non-friable, and has good commercial properties, which can help promote the selection of good varieties of *M. esculenta* and provide a theoretical basis for their cross-breeding.

**Figure 2:**
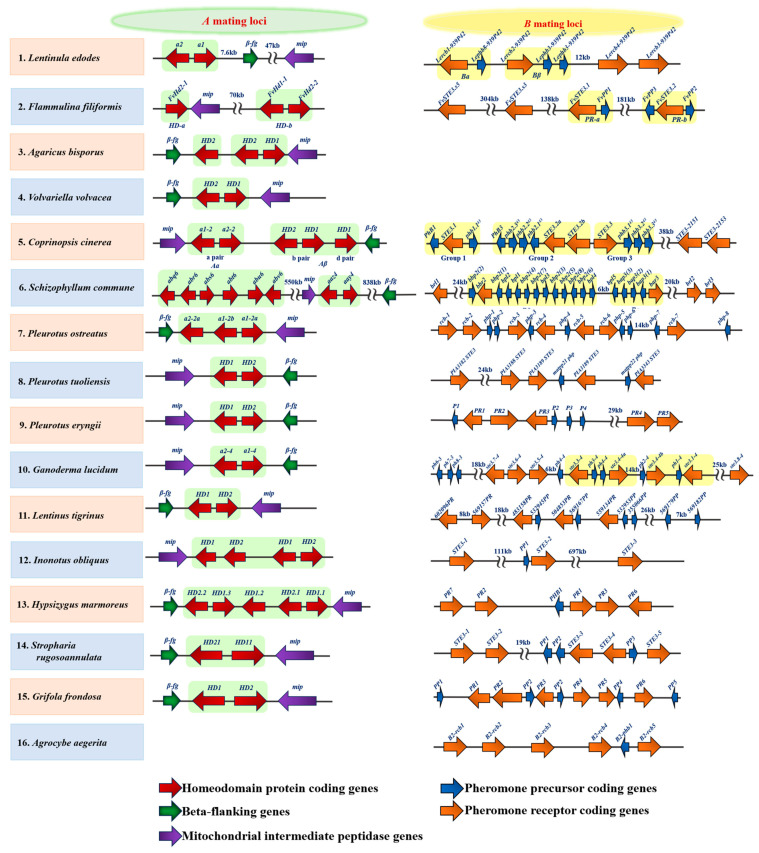
Schematic diagram showing the molecular genetic structure of mating loci in common mushrooms [120,121,122,123,124,125,126]. 1. *Lentinula edodes* ‘L54A’ (*A* locus) and ‘No. 939P42’ (*B* locus); 2. *Flammulina filiformis* ‘KACC42780’; 3. *Agaricus bisporus* ‘H97’; 4. *Volvariella volvacea* ‘V23-1’; 5. *Coprinopsis cinerea* ‘Okayama 7 #130’; 6. *Schizophyllum commune* ‘H4-8’; 7. *Pleurotus ostreatus* ‘PC15’; 8. *Pleurotus tuoliensis* ‘489P1’; 9. *Pleurotus eryngii* ‘181’ (*A* locus) and ‘SDF55’ (*B* locus); 10. *Ganoderma lucidum* ‘G.260125-1’; 11. *Lentinus tigrinus* ‘Lenti7’; 12. *Inonotus obliquus* ‘MDJCBS88’; 13. *Hypsizygus marmoreus* ‘H3-1’; 14. *Stropharia rugosoannulata* ‘SR1’; 15. *Grifola frondosa* ‘y59’; 16. *Agrocybe aegerita* ‘YS8’. The mating loci and the mating genes in the figure are named according to the literature.

Basidiomycota comprise the majority of edible and medicinal fungi and are complex mating-type systems composed of multiple loci and multiple alleles, which are usually divided into dipolar and tetrapolar mating systems, accounting for 25% [115] and 55–65% [116], respectively. The most typical of these is the latter, which contains two unlinked *A* mating-type and *B* mating-type loci. According to edible and medicinal fungi genetics, the *A* mating-type loci contain *HD1* and *HD2* genes encoding homology domain transcription factor proteins, which control behaviors such as the production of clamp connections, nucleus pairing, and division of the nucleus [117,118], whereas the *B* mating-type loci encode pheromone receptors and pheromone precursors, which control behaviors such as the migration of the nucleus and ablation of septa between cells [119]. The structure of the mating-type loci of common edible and medicinal fungi is shown in Figure 2 [120,121,122,123,124,125,126], which shows that the *A* mating-type loci contain a pair of mating-type genes that encode a pair of dissimilar homozygous proteins—HD1 and HD2; only at different alleles can they be paired to form heterodimers, whereas the *B* loci contain a pheromone receptor-coding gene and one to several pheromone-coding genes and originate from different alleles before they can recognize each other. From the perspective of collinearity, most of the *A* mating-type loci follow the typical *mip-HD-β-fg* structure, but *L. edodes* and *F. filiformis* do not, and the order and number of genes present at the loci vary considerably in different species, which possesses the characteristics of mating-type locus polymorphism. With the continuous evolution of nature, certain mating genes will be added or lost; separate *HD* genes will exist at the *A* locus of *F. filiformis*, *A. bisporus*, *C. cinerea*, *S. commune*, *P. ostreatus*, etc.; and a few pheromone precursor coding genes will exist outside the *B* locus. The mating of compatible monokaryons carrying mating genes *A* and *B* is necessary for the formation of fruiting bodies. The *A* mating genes *HD1* and *HD2* and the *B* mating genes *PEphb3.1* and *PEphb3.3* from the monokaryon of *P. eryngii* were knocked into compatible monokaryotic strains by our team using the PMT technique [120]; the obtained transformants had clamp connections and distinct dikaryotic mycelium cells, which is conducive to the further exploration of mating-type gene function. Subsequently, the team knocked *A* and *B* mating genes into the mononucleosomes of *P. eryngii* and reported that they could promote the development of the fruit body but could not form basidiospores. Transcriptomic analyses showed that the transformants activated the expression of endogenous homology domains, pheromone receptor genes, and mating-type signaling pathways, providing new avenues for the study of fruiting body development [127].

Mating-type genes are important functional genes that regulate the development of mycelia and fruiting bodies in dikaryotes and have a complex mating-type system that should be widely considered. A comprehensive understanding of the mating loci and genetic polymorphisms of strains is of great benefit for species maintenance, variety innovation, strain identification, and molecular breeding. However, there are still significant gaps in the field of mating-type genes, such as insufficient knowledge of the *HD* genes alone in the *A* locus of edible and medicinal fungi and the pheromone precursor coding genes outside the *B* locus, which need to be further studied in depth by resolving methods such as gene insertion and knockout. The evolutionary process and causes of the alteration of chromosome structure as a result of the covariation of mating-type genes still have to be investigated in detail, and it is necessary to identify the effect of synthetic pheromone peptides or products secreted through the autoregulation of clamp cell formation [128].

### 3.2. Research Progress on Genes Related to Mycelium and Fruiting Body Development

Since the middle of the 20th century, the mechanisms of fruiting body growth and development have become the focus of research on edible and medicinal fungi. As the most complex structure in fungi, the growth and development of fruiting bodies are synergistically regulated by environmental factors such as temperature, humidity, light, and CO_2_ concentration and intrinsic factors dominated by genes. The growth and development stages involve the regulation of a variety of pathways, including signaling, nutrient synthesis and transport, metabolism, hormones, and transcription.

Currently, the functions of more than 40 genes related to mycelial growth and fruiting body development of edible and medicinal fungi have been analyzed and verified, mainly in commonly cultivated species such as *F. filiformis*, *G. lucidum*, *P. ostreatus*, *C. militaris*, and *V. volvacea* (Table 3). Among them, regulatory genes can be mainly divided into coding genes, such as hydrophobin, lectin, transcription factor, and laccase, which play crucial roles in the different growth and developmental stages of edible and medicinal fungi during their complete life histories.

Hydrophobic proteins are involved in multiple developmental processes in edible and medicinal fungi and play an important role in spore spread, pathogenesis, and the formation of ectomycorrhiza. Tao et al. [136] used interference methods to identify the function of *Hyd9* in *F. filiformis* and reported that the surfaces of the aerated mycelia were dense after overexpression, while the surfaces of the silenced mycelia were sparse, suggesting that the hydrophobic protein could help fungi form aerated mycelia. Han et al. [129] knocked out the *vmh2* and *vmh3* genes in *P. ostreatus* and reported that the strain grew slower than the wild type under the stressed conditions of SDS and H_2_O_2_. However, for *C. militaris*, the class II hydrophobic protein-coding gene *Cmhyd1* and the class I hydrophobic protein-coding gene *Cmhyd4* played opposite genetic functions. Li et al. [151] constructed a *Cmhyd4* knockout vector, and the deletion of this gene increased the density, height, and conidium production of the strain compared with the wild type and negatively regulated the development of the fruiting body of *C. militaris*.

Lectins, as non-immunoglobulins, play an important role in the growth and development, morphogenesis, and molecular recognition of edible and medicinal fungi during the early stages of mycorrhizalization and nutrient storage. Su et al. [130] cloned *polectin2* from *P. ostreatus*, successfully constructed a recombinant expression vector, conducted low-temperature stress experiments on a positive transformer, and reported that the gene has a low-temperature tolerance function. Lu et al. [137] used RNA interference and the overexpression of *Fv-JRL1* in *F. filiformis* and reported that this gene plays a positive role in the formation of aerogenic mycelia and fruiting bodies. Our team cloned the *CMlec3* gene in *C. militaris*, successfully expressed it heterologously in *E. coli*, and reported that the expression level of this gene in the primordium was 5.19 times that in the mycelium and 1.35 times that in the fruition body, indicating that this gene is related to fruiting body development [152].

Transcription factors are important protein molecules involved in transcription initiation and play important roles in mycelial growth, fruiting, spore formation, sexual reproduction, and sclerotium formation in edible and medicinal fungi [168]. Lyu et al. [138] conducted knockout and overexpression experiments on *Fvclp1* in *F. filiformis* and reported that the knockout strain produced fewer fruiting bodies than the wild type, and the overexpressed strain had improved fruiting ability. This indicates that *Fvclp1* can effectively promote sexual reproduction and fruiting body development. Wu et al. [140] verified the function of *lfc1* in *F. filiformis* and reported that the strains overexpressing *lfc1* had reduced buttons and folds on the edge of the cap, and the stalk length of the strains with low *lfc1* increased by more than 27%. These results suggest that the goals of shortening cultivation times and increasing yields could be achieved by reducing the expression of *lfc1*. Zhang et al. [149] silenced *GlSwi6* in *G. lucidum* and observed that the mutant strains exhibited multiple developmental defects, such as reduced mycelial growth and the formation of fruitless bodies. Chen et al. [160] overexpressed the *Vvrin1* gene of *V. volvacea*, and the transformed hyphae obtained were thicker. The surface color of the colony was darker, indicating that the gene regulates the mycelial growth rate and pigment synthesis or accumulation in this species.

Laccase is a copper-containing polyphenol oxidase that has a strong ability to degrade lignin and catalyze the oxidation of various substrates. Most edible and medicinal fungi are capable of secreting laccases during growth and development, thus effectively utilizing lignin in the substrate. Armas-Tizapantzi et al. [134] silenced *lacc2* in *P. ostreatus* and reported that laccase activity decreased by 30–55%; mycelium development was delayed in PDA medium compared to the wild type; and air mycelia, primary bases, and fruity bodies could not be formed in wheat straw cultures. Zhang et al. [155] cloned and overexpressed *lcc1* in *H. marmoreus* and reported that the transformed mycelia grew faster, the fruit body matured approximately five days earlier than the wild type, and the laccase activity was higher during development. These results suggest that *lcc1* plays a positive role in regulating the growth and development of mycelia and fruit bodies.

Currently, there are many studies on the regulatory genes of mycelial and fruiting body growth and development in edible and medicinal fungi, but the similarity of the same family of genes from different species is quite different. Most studies have focused only on whether and how many genes are expressed at different stages of fruiting body growth and development, and there are few studies on the response mechanisms of various genes and environmental factors. The development and maturation of edible and medicinal fungi are adaptations to matrix carbon and nitrogen sources, pH, humidity, temperature, and other environmental factors. Therefore, combined with the study of the regulatory mechanisms, we can better understand the functions of genes related to the development of edible fungal mycelia and fruiting bodies.

### 3.3. Research Progress on Genes Related to Substrate Utilization and Nutrient Transport

Large amounts of rice, wheat, corn straw, and other agricultural by-products are produced worldwide every year, which are rich in cellulose and hemicellulose for resource utilization. Further enhancing the hydrolysis ability and saccharification efficiency and solving the inhibition of complex products has become an important issue in the field of substrate utilization by edible and medicinal fungi. Li et al. [169,170] expressed *LeXyn* and *LeCel7A*, the xylanase-encoding genes of *L. edodes* in *P. pastoris*, and reported that the two recombinant proteins enhanced the hydrolyzing ability of rice straw, wheat straw, and corn straw. The team further reported that *L. edodes* xylanase could effectively hydrolyze wheat and simultaneously resist pepsin degradation at the same time, which could improve the utilization rate of wheat in animal feed [171]. To improve the cold adaptability of mesophilic cellulase, our team isolated a novel cold-active cellobiohydrolase-coding gene *vvcbh1-1* from *V. volvacea* and successfully expressed it heteroglyphically in *T. reesei* after low-temperature induction, which is beneficial for the efficient low-temperature storage of straw [172]. Li et al. [173] successfully expressed the new lytic polysaccharide monooxygenase coding gene *PdLPM09A* from *Pleurotus djamor* in *P. pastoris*, and when the dosage of rPdLPM09A was 0.66 mg/g and hydrolyzed for 48 h, the greatest improvement in cellulase-mediated saccharification of corn stalk was achieved. The product inhibition effect of the cellulose hydrolysis process is an important problem to be solved in cellulase preparations. Our team codon optimized and heterologously expressed the endoglucanase-encoding gene *pacel3a* from *Polyporus arcularius* in *T. reesei*; when compared with the natural PaCel3A, the recombinant protein CMCase had higher activity and the glycosylation efficiency was increased by 17%, which effectively mitigated the product inhibition of the cellulase complex of *T. reesei* [174].

Forest ecosystems play an important role in the global carbon cycle, where edible and medicinal fungi degrade persistent organic compounds in soil litter and humus layers. Heneghan et al. [175] silenced the serine protease-coding gene *SPR1* in *A. bisporus* and reported that *SPR1* contributed to nutrient acquisition in compost and was a key enzyme in the biomass degradation process. Jiang et al. [176] analyzed the high-quality genome of *G. lucidum* and reported that *salh*, *phea*, *cyp53a1*, *cyp102a*, *glpk*, and *amie* in *G. lucidum* are enriched in functions such as plant–pathogen interactions and the benzoate degradation pathway, which provides valuable genomic resources for the utilization of conifer cultivation substrates.

Edible and medicinal fungi are rich in the high-quality proteins and amino acids required by humans, and their content and composition are important indicators for evaluating the nutrition and flavor of edible and medicinal fungi. Lian et al. [177] silenced the alkaline leucine-transcription-factor-coding gene *Gcn4* in *G. lucidum* and reported that the amino acid content of the mutant strain was reduced by 54.2–88.3% compared with the wild type. They hypothesized that *GCN4* could modulate the expression of amino-acid-related synthesis genes and transporter genes, providing precursors for amino acid biosynthesis by enhancing the TCA cycle and glycolytic pathway. Carbon Catabolite Repressor (CCR) directly affects the selective utilization of complex carbon sources by fungi. Pareek et al. [178] used the RNPs system to knock out *cre1*, an important transcription-factor-encoding gene of CCR in *C. cinerea* and reported that the gene expression profile of the mutant showed dysregulation of transcription-factor-encoding genes, such as carbohydrate metabolism, plant-cell-wall-degrading enzymes, and plasma membrane transporters, which provided gene recognition targets for related transcription factors in the process of wood degradation by edible and medicinal fungi.

### 3.4. Genes Related to Environmental Response

In addition to the genetic background and culture medium, the growth environment is a key factor affecting the fruiting body development of edible and medicinal fungi, having impacts on important traits such as shape, yield, flavor, and stress resistance. Exploring the response mechanisms of edible and medicinal fungi to environmental factors, such as temperature, humidity, light, and carbon dioxide concentration, can help enhance cultivation environment control and, thus, improve the yield and quality of germplasm. As shown in Table 4, the functions of environmental-response-related genes in edible and medicinal fungi are mainly related to temperature, light, and metal stress.

Temperature plays an important role in fruiting body differentiation in edible and medicinal fungi. Yang et al. [179,181] conducted heat and cold stress treatments on *V. volvacea*, analyzed the enzyme activities and growth of the strains, and reported that *VvCAT1* had a defense response function under low-temperature stress and *Mn-SOD* could tolerate heat, cold, and salt stress, providing a theoretical basis for the anticlimactic-directed breeding and preservation of *V. volvacea*. In addition, researchers have reported that *LeMnP1*, *LeDnaJ*, *YUCCA8*, *TrpB*, and *LetrpE* in *L. edodes* are beneficial for enhancing the heat tolerance of mycelia and improving the defense response to heat stress. Over the course of long-term evolution, edible and medicinal fungi have developed a perfect photosensory system to better adapt to the environment. For example, the blue light receptor coding gene *Cmwc-1* in *C. militaris* contributes to pigment precipitation and promotes conidiation [182], the blue light complex transcription factor coding genes *Wc-1* and *Wc-2* in *S. commune* inhibit vegetative growth and protect against phototoxicity [183], and blue light receptor coding gene *Icwc-1* in *Isaria cicadae* has the function of regulating genes related to pigment and enzyme synthesis [184]. Similarly, the blue light and its receptor white collar complex coding genes *FfWc1* and *FfWc2* in *F. filiformis* can regulate the role of morphogenesis [185]. In addition, *Gf.BMR1* from *G. frondosa* contributes to fruiting body development and pigment accumulation [186], *abl-D* from *L. edodes* promotes the formation of brown film in mycelia [187], and *Pabs* from *A. bisporus* [188] enhances UV tolerance. These studies can provide references for light control in the production of edible and medicinal fungi in factories. Since edible and medicinal fungi can accumulate various trace elements in the cultivation matrix, excessive heavy metals have become a food safety issue for consumers. *M. esculenta* is a rare edible fungus that needs to be cultivated in soil. However, heavy metal pollution in the soil poses a potential threat to its mycelial growth and the quality and safety of fruiting body products. Chen et al. [189] reported that *ATX1*, a keto chaperone gene, may play an important role in response to Cd stress in *M. esculenta*, which is of great significance for revealing the molecular mechanisms of Cd metabolism and the reduction of Cd in edible and medicinal fungi. Bian Yinbing’s team analyzed the molecular mechanism of cadmium tolerance differentiation in *L. edodes* using combined mRNA and milRNA analysis and verified the role of α amylase gene *LeAmy* in enhancing the cadmium tolerance of *L. edodes* [190]. This lays an important theoretical foundation for the selection of excellent *L. edodes* varieties that are suitable for heavy metal remediation.

### 3.5. Research Progress on Genes Related to the Synthesis and Regulation of Important Active Substances in Edible and Medicinal Fungi

Over the course of long-term evolution and environmental adaptation, edible and medicinal fungi have developed efficient chemical defense mechanisms and produced metabolites with novel structures and diverse activities. Based on their chemical structures, these can be divided into polysaccharides, terpenes, phenols, and nitrogenous heterocyclic compounds and their derivatives. Many of these compounds have been proven to have immunomodulatory, anti-tumor, antibacterial, antiviral, antioxidant, antihypertensive, and antilipemic properties [191]. The analysis of the biosynthetic regulatory genes in the metabolic pathways of the active metabolites from edible and medicinal fungi helps to achieve the targeted synthesis of target molecules using synthetic biology technologies and provides a new method for the cultivation of new varieties and processed products with special nutrition, flavor, and health effects in the edible and medicinal fungi industry.

#### 3.5.1. *Ganoderma* Triterpenoids

*G. lucidum* is an important medicinal and food-homologous fungus. Various triterpenoids have been isolated from *G. lucidum* spore powder, fruiting bodies, mycelia, and fermentation broth extracts, which have a wide range of pharmacological activities, such as anti-tumor [192], antivirus [193], anti-inflammatory [194], liver protection, and detoxification activities [195], and ameliorating the psychological effects of anxiety and depression [196].

**Figure 3:**
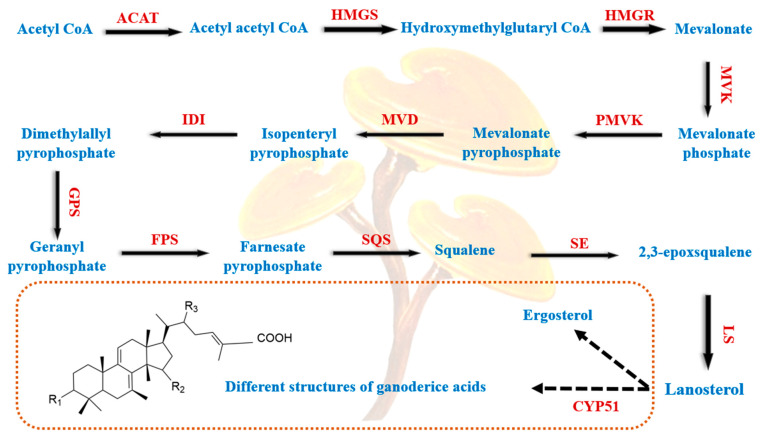
Triterpenoid biosynthesis pathway in *Ganoderma* [197,198,199]. ACAT: Acelyl-CoA acelyltransferase; HMGS: 3-hydroxy-3-methylglutaryl-CoA synthase; HMGR: 3-hydroxy-3-methylglutaryl-CoA reductase; MVK: mevalonate kinase; MVD: Pyrophosphomevalonate decarboxylase; IDI: isopentenyl diphosphate isomerase; FPS: farnesyl pyrophosphate synthase; GPS: Geranyl pyrophosphate synthase; SQS: Squalene synthase; SE: Squalene epoxidase; LS: lanosterol synthase; CYP51: sterol 14a-demethylase.

The biosynthesis of *Ganoderma* triterpenoids can be divided into two parts—the synthesis of lanosterol and the biosynthesis of different ganoderic acid monomers (Figure 3) [197,198,199]. Lanosterol is synthesized via the mevalonate pathway (MVA), which is followed by a series of reactions to produce lanosterol-containing triterpenes and ganoderic acid monomers with different structures. Currently, *Gl-acat*, *Gl-hmgs*, *Gl-hmgr*, *Gl-mvd*, *Gl-fps*, *Gl-sqs*, and *Gl-ls* are the key genes cloned in the triterpene biosynthesis pathway of *G. lucidum*, and their gene expression levels are consistent with triterpene content [197]. In addition, the biological regulatory factors of *Ganoderma* triterpenoids include Ca^2+^ signaling and plant hormones, such as salicylic acid (SA) and methyl jasmonate (MeJA), which regulate the biosynthesis of *Ganoderma* triterpenoids and CYP450 inducers [200].

Ganoderic acid is a highly oxidized secondary metabolite of lanosterane-type tetracyclic triterpenoids—classified into type I and type II ganoderic acid—and is an important indicator for controlling the quality of *G. lucidum*. Thus, the study of ganoderic acid synthesis genes can help the breeding and creation of high-quality *G. lucidum* varieties. The structural genes that have been cloned and reported in the process of ganoderic acid biosynthesis include *ACAT* [201], *HMGS* [202], *HMGR* [60], *FPS* [203], and *MVD* [204]. The high and low expression levels of these genes are consistent with the trends of ganoderic acid content, and they are directly involved in the process of ganoderic acid synthesis. Jiang et al. [205] screened cytochrome *P450* genes related to ganoderic acid biosynthesis and reported that *cyp512v2* played an important role in ganoderic acid T biosynthesis. In addition to the structural genes, regulatory genes in the ganoderic acid biosynthesis pathway directly regulate catalase (Table 5). From Table 5, it can be seen that the regulation of ROS signaling and Ca^2+^ signaling affects the synthesis of ganoderic acid, and the changes in ROS content caused by silencing *AOX*, *GCN4*, and *GlSnf1* are consistent with the changes in ganoderic acid content, while the silencing of *GlTert* causes an increase in the ROS content but a decrease in ganoderic acid content. These specific synthetic regulatory mechanisms need to be further explored.

The biosynthesis of *Ganoderma* triterpenoids and their regulatory mechanisms have a profound impact on further improving the medicinal value of *G. lucidum*. The simple genetic operation systems and gene-editing technologies applied to *G. lucidum* provide a good platform for the synthesis of important active substances. For example, a high-throughput automated screening platform can identify key genes in the triterpenoid biosynthesis pathway of *G. lucidum* to achieve efficient heterologous biosynthesis [109], increase yield by adjusting the copy number of plasmids [213], and solve the problem of poor fitness of homologous gene expression and low yield by a closely related host [214].

#### 3.5.2. Cordycepin

The structural formula of cordycepin, C_10_H_13_N_5_O_3_, chemically known as 3′-deoxyadenosine, was first isolated from the fermentation broth of *C. militaris* by the German scientist Cunningham in 1950 [215]. As an important secondary metabolite in *C. militaris*, it has anti-tumor [216], neuroprotective [217], antibacterial, and anti-inflammatory effects [218], and alongside other functions, improves the body’s comprehensive resistance to disease [219].

**Figure 4:**
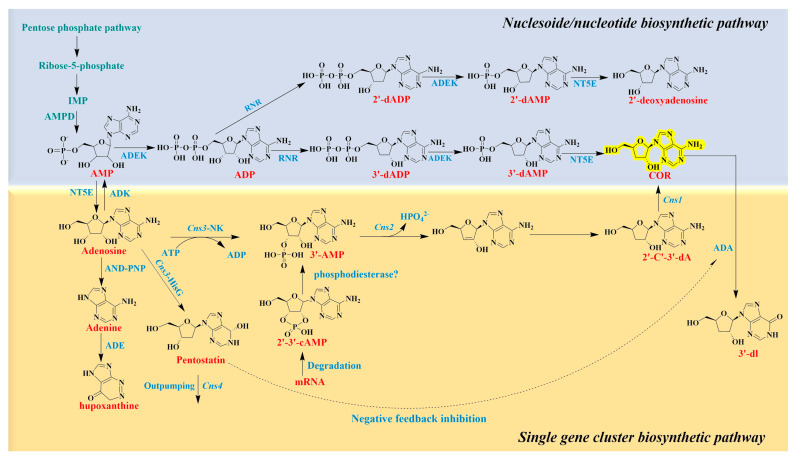
Map of the cordycepin biosynthesis pathway [220,221,222]. IMP: inosine monophosphate; 3′-AMP: adenosine-3′-monophosphate; 2′-C-3′-dA: 2′-carbonyl-3′-deoxy adenosine; 3′-dI: 3′-deoxyinosine; COR: cordycepin; AMP: adenosine monophosphate; ADP: adenosine diphosphate; dAMP: deoxyadenosine monophosphate; dADP: deoxyadenosine diphosphate; RNRs: ribonucleotide reductases; ADK: adenosine kinase; ADEK: adenylate kinase; NT5E: 5′-nucleotidase; ADA: adenosine deaminase; NK: an N-terminal nucleoside kinase; HisG: a C-terminal HisG family of ATP phosphoribosyltransferases.

With the completion of whole-genome sequencing and data analysis, the biosynthetic pathway of cordycepin was gradually clarified; it is first converted from the pentose phosphate pathway to AMP and then synthesized through the nucleoside/nucleotide biosynthetic pathway and single gene cluster biosynthetic pathway (Figure 4) [220,221,222]. AMP generates ADP under the action of ADEK and then generates 3′-dADP and 2′-dADP under the action of ribonucleotide reductases. 3′-dADP generates 3′-dAMP under the action of ADEK, followed by cordycepin under the action of NT5E, while 2′-dADP finally generates 2′-deoxyadenosine to participate in nucleotide metabolism. For single gene cluster synthesis, 3′-AMP is the precursor of cordycepin synthesis, which is mainly converted by the nucleotide domain of *Cns3*-NK and is produced by the phosphorylation of 2′,3′-cAMP, which is subsequently converted via *Cns2* and *Cns1* redox to cordycepin. 

Xia et al. [223] identified four highly homologous protein-coding genes (*Cns1*, *Cns2*, *Cns3*, and *Cns4*) in *C. militaris*. Protein function analysis revealed that *Cns1* encodes oxidoreductase, *Cns2* encodes ion-dependent hydrolase, *Cns3* encodes ATP-dependent phosphotransferase, and *Cns4* encodes an ABC-type transporter protein. To further determine the role of these functional genes, *Cns1*–*Cns4* knockout and heterologous expression in *S. cerevisiae* showed that *Cns1* and *Cns2* are key genes for cordycepin biosynthesis, *Cns3* is involved in the biosynthesis of pentostatin, and *Cns4* may encode the pentostatin transporter. However, pentostatin can prevent cordycepin deamination by inhibiting ADA activity, which is a key factor in regulating cordycepin biosynthesis. SNF1/AMPK are important regulators of fungal development and metabolism [224]. Our team increased the expression level of the cordycepin biosynthesis gene cluster by knocking out SNF1/AMPK in *C. militaris*, which resulted in more than a seven-fold increase [225]. In the same year, Chen et al. [226] overexpressed two transcription factors, *CmTf1* and *CmTf2*, in *C. militaris*, which resulted in a three-fold increase in cordycepin content, up to 99 mg/L after liquid fermentation, compared to that in the wild-type. Studies have shown that improving the hypoxic adaptability of the fungus contributes to the synthesis of the active substance. For example, our team overexpressed the predicted bHLH transcription factors of sterol regulatory element binding proteins (SREBPs) in vivo, which increased the cordycepin content more than two-fold, providing a scientific basis for optimizing the cultivation and fermentation gas supply strategy [227].

Synthetic biology has developed rapidly in recent years, with prokaryotic and eukaryotic chassis cells being used to express exogenous genes, realizing the efficient production of a wide range of active substances. During the long-term evolution of *C. militaris*, through the introduction of efficient cellular-degrading enzymes, this species has acquired the ability to degrade waste bacterial residues, and the genes related to the cordycepin synthesis gene cluster have been knocked out. This has continuously improved the production of pentastatin anti-cancer drugs and finally realized environmentally friendly and sustainable green bio-manufacturing [228]. In addition, the long biosynthetic cycle of natural cordycepin and competition with other intracellular secondary metabolites for substrates are the main factors restricting the industrial production of cordycepin. Therefore, it is necessary to focus on the transcription factors, cofactors, and key enzymes involved in metabolic processes. To continuously expand the scale of cordycepin production, optimizing the metabolic flux of the chassis, reducing the cytotoxicity of the product, and improving the efficiency of extracellular transport are required.

#### 3.5.3. Polysaccharides

The polysaccharides of edible and medicinal fungi are natural macromolecular active substances that have a variety of effects, such as antibacterial, antiviral, anti-cancer, antioxidant, and anti-diabetic properties. For example, the polysaccharide–protein complex of *Phellinus igniarius* can be used as an anti-inflammatory agent [229], *L. edodes* polysaccharides can reduce the proliferation of leukemia cells [230], and *M. esculenta* polysaccharides can regulate the activity of antioxidant enzymes [231]. Due to their good biological activity, polysaccharides from edible and medicinal fungi can be used as food additives, biodegradable films, functional foods, nutritional supplements, and animal feeds after processing. Furthermore, owing to their anti-aging and antioxidant effects, they can be used in functional cosmetics, such as *L. edodes* polysaccharides, which can be used as a facial cream ingredient with excellent moisture absorption, good water solubility, and viscosity [232].

Functional analysis of polysaccharide synthesis genes in edible and medicinal fungi will help to enhance the content of target products and promote the application of edible fungal polysaccharides in food, cosmetics, and environmental protection. Polysaccharides from edible and medicinal fungi can be divided into intracellular polysaccharides (IPS), extracellular polysaccharides (EPS), and cell wall polysaccharides (CWP). Currently, IPS and EPS are primarily observed in *C. cinerea*, *C. militaris*, *G. frondosa*, *G. lucidum*, and *Hericium erinaceus*. Zhou et al. [233] overexpressed phosphoglucomutase (PGM) and UDP-glucose pyrophosphorylase (UGP) genes in *C. cinerea* singly or with co-overexpression and reported that the overexpression of *PGM* and *UGP* increased the EPS content by 30% and 16%, respectively, and the co-overexpression of *PGM* and *UGP* increased the EPS content by 75%. These results suggest that compared to a single pathway, multiple-gene co-overexpression increases polysaccharide production compared to single pathways. Similarly, the homologous overexpression of *C. militaris PGM* showed that the engineered strain produced 78.13% more EPS than the wild-type strain [234]. Cui et al. [235] silenced the glucan synthase gene *GFGLS* in *G. frondosa* and reduced EPS production by 0.38 g/L. Subsequently, our team silenced the two glucan synthase genes, *GFGLS* and *GFGLS2*, in *G. frondosa* and reported that the silencing of *GFGLS2* synthesized less EPS than that of *GFGLS* and that their co-silencing had a synergistic downregulation effect that slowed down the production of glucan [236]. Zan et al. [237] constructed an RNA interference vector of the reverse-double promoter system of *gfugp* in *G. frondose*, in which the EPS was mainly composed of glucose (98.25%) and a small amount of arabinose (0.86%); silencing *gfugp* reduced the glucose content by 62.79–66.79%, and it was speculated that the silencing of *gfugp* affects the synthesis of EPS. Xu et al. [238] overexpressed *PGM* in *G. lucidum*, and IPS and EPS increased by 40.5% and 44.3%, respectively, compared to the wild type. Li et al. [239] introduced the *Vitreoscilla hemoglobin* coding gene *VHb* into *G. lucidum* and reported that the coding genes of polysaccharide biosynthesis enzymes, such as *PGM*, *UGP*, and *GLS*, were affected, which increased the IPS and EPS by 30.5% and 88.2%, respectively, compared with the wild type. This could be beneficial for large-scale fermentation by improving the potential application of *G. lucidum* polysaccharides. Xu et al. [240] co-overexpressed β-1,3-glucan synthase (*gls*) and UDP-glucose pyrophosphorylase in *G. lucidum*, and IPS and EPS were 1.31 times and 1.50 times higher than in the wild type, which is helpful for the ultra-production and application of *G. lucidum*. Our team used *E. coli* and *S. cerevisiae* to validate the function and activity of A6180, the proposed gene encoding UDP-Glucose 4-Epimerase (*UGE*) in the highly active polysaccharides of *H. erinaceus*, and reported that the recombinant protein converted UDP-α-D-glucose to UDP-α-D-galactose under optimal conditions and enhanced macrophage activity in vitro [241].

Currently, polysaccharides from edible and medicinal fungi are mainly extracted from cultivated and wild-picked mushrooms; however, the production of polysaccharides through genetic or metabolic engineering has certain limitations, including unstable strains, unknown side effects, complex downstream extraction processes, and unclear assembly and polymerization processes of synthetic precursor nucleotide glycosaminoglycan. The polysaccharides of some edible and medicinal fungi, such as *G. frondose* protein binding β-glucose [242], *Coriolus versicolor* protein binding polysaccharide PSK [243], and *S. commune* neutral polysaccharide SPG [244], can be used as anti-tumor, anti-cancer and immunomodulatory drugs [245]. In addition, other bioactive polysaccharides still need to be further studied, and the positive impact of genetic engineering on polysaccharide production of edible and medicinal fungi will greatly promote the potential application of in vitro synthesis-related research in industrial production.

#### 3.5.4. Immunomodulatory Proteins of Edible and Medicinal Fungi

Fungal immunomodulatory proteins (FIPs) are active compounds isolated from edible and medicinal fungi. To date, more than 30 types of FIPs have been identified in edible and medicinal fungi, such as *F. filiformis*, *A. camphorata*, *M. esculenta*, *G. lucidum*, and *H. marmoreus*, with coagulation, immune-system-regulation [246], and anti-inflammatory [247], anti-allergic, and anti-tumor effects [248]. With the development of biotechnology, at least 26 recombinant FIPs (rFIPs) related to edible and medicinal fungi have been identified using genome mining, homologous cloning, and protein purification to form a unique protein family [249].

Lin et al. [250] cloned FIP-fve from *F. filiformis* and expressed it in *P. pastoris* GS115; the results showed that the purified FIP-fve protein had immunomodulatory and anti-tumor activities in vitro activity assay. Li et al. [251] efficiently expressed the immunomodulatory protein ACA from *A. camphorata* through *E. coli* with a yield of up to 187.122 μg/mL, and the purified rACA significantly promoted the proliferation and phagocytic activity of mouse macrophages, which can be used as a food additive and immunomodulator. Wu et al. [252] identified a new FIP-mAb in *M. esculenta* and successfully expressed this in *P. pastoris* followed by apoptosis experiments. They showed that rFIP-mco reduced inflammatory cytokines by inhibiting NF-κB signaling, which has broad application prospects in cancer treatment and medical research. Zhou et al. [253] cloned *FIP-gap1* and *FIP-gap2* from *G. lucidum* and successfully expressed them in *P. pastoris*, and the recombinant contents reached 247.4 mg/L and 197.5 mg/L, respectively, and both rFIPs were able to coagulate blood and enhance IL-2 and IFN-γ in vitro bioactivity assay. Our team conducted considerable research in this field. Seven FIPs were identified and their physiological and biochemical characteristics were predicted in *V. volvacea*, and it is speculated that the new FIP-vvo82 had efficient immunomodulatory activity for inducing spleen lymphocytes [254]; two immunomodulatory proteins, LZ-8 and LZ-9, in *G. lucidum*, were predicted and analyzed comparatively using bioinformatics and the immunomodulatory activity of LZ-8 was analyzed from the perspective of molecular structure [255], and the amino acids closely related to high activity were observed for subsequent research [256]; two immunomodulatory proteins, FIP-lti1 and FIP-lti2, derived from *L. tigrinus*, were identified, and their expression and purification revealed that they could protect the liver from Concanavalin A-induced hepatic oxidative damage through the Nrf2/NF-κB/NLRP3/IL-1β pathway [257]; and a novel FIP-hma was observed in *H. marmoreus* and its expression in *E. coli* resulted in the upregulation of iNOS, IL-264, IL-7β, and TNF-α levels by rFIP-hma in macrophages, which is hypothesized to activate immune responses by regulating central cytokines and provides new ideas for the study of the physiological functions of FIPs and their applications in the field of medicine [258].

Recombinantly expressed FIPs obtained by genetic engineering have similar structures and biological functions to those obtained by natural isolation and purification and have better heat and acid resistance and dehydration stability, which can potentially be exploited as anti-cancer drugs. However, further research is required on the mechanisms and reasons for the differences in the activities of FIPs to improve their pharmacological activities and optimize recombinant expression conditions to obtain the highest yields, which will continue to support application in the fields of food and medicine.

## 4. Conclusions and Prospects

Edible and medicinal fungi can synthesize a variety of natural products, such as antibiotics, pigments, enzyme inhibitors, and hormones, and are widely used in medicine, the chemical industry, agriculture, and basic biological research. However, their complex genetic backgrounds hinder further development and utilization. In recent years, researchers have continuously strengthened and developed efficient chassis cells and multi-omics technology platforms, constructed efficient genetic expression platforms and developed bidirectional selection marker systems. With the combination of high-precision gene-editing technology, remarkable achievements have been made in the study of the mating type, fruiting body development, substrate utilization, nutrient transport, environmental responses, and important active substance synthesis regulatory genes in edible and medicinal fungi. However, scientific problems still urgently need to be resolved.

First, regarding functional analysis technologies for edible and medicinal fungi, the lack of high-quality reference genomes is an important reason for the slow pace of research. Therefore, it is necessary to strengthen standardized, versatile, and accurate genotype and phenotype identification platforms and bioinformatics analysis platforms, conduct in-depth pan-genome sequencing and analysis, continuously explore new genes, and find excellent alleles. In this process, due to the spread of edible and medicinal fungi all over the world, the corresponding strain and species names have not been unified. We hope that the unification of database nomenclature can be strengthened in the future.

Second, the structure and genes of mating-type loci in edible and medicinal fungi are polymorphic, and the collinearity of mating-type loci in different species varies greatly; therefore, the evolutionary process needs to be further studied. Further studies on photoreceptor proteins, signaling pathways, fruiting-body-induced temperature sensor systems, and environmental response mechanisms are needed. Through the further analysis of functional gene regulatory networks and related gene clusters, omics technology can be combined with metabolic network research to promote further developments in bioengineering [259].

Finally, with the continuous development of synthetic biology, the application of functional gene research in various fields should be strengthened. Future studies should reveal whether SMS can be transformed into a pure mycelic material, which can then be used to produce materials with both compressive strength and flexibility for the packaging and construction industries. In addition, mating-type genes can be used for precise and efficient breeding, and stress resistance genes can be used to improve fruiting body yield, thus moving towards an era of precision molecular breeding. Established genetic transformation and gene-editing technologies can be used to study the mechanisms of degeneration, and high-quality engineered strains can be constructed by crossing genes containing opposite mating types. Furthermore, edible and medicinal fungi have great medicinal potential and are valuable to human health. In the future, the development of the edible and medicinal fungi industry will be upgraded to the field of intensive processing and health, such as the expression of key genes in the active substance synthesis pathways of uncultivable edible and medicinal bacteria in heterologous hosts and their application to large-scale production in cell factories.

## Figures and Tables

**Table 1:** General methods for genetic transformation of edible and medicinal fungi.

Method	Advantage	Disadvantage	Application Case	Refs.
ATMT	Efficient; copy number of DNA is inserted low; high efficiency of homologous recombination	Time-consuming; partially sensitive to acetosyringone	Construction of ATMT in *H. marmoreus*, with *CaMV35s* as a promoter to efficiently drive the expression of *GUS* genes	[37,38]
PMT	Efficient; high copy number of inserted DNA; suitable for most edible and medicinal fungi	Requires special lytic enzyme; difficult to prepare and not easy to store; low regeneration efficiency	Successful PMT of linear DNA fragments containing the bar gene into *C. militaris* protoplasts yielded 87 positive mutants containing the bar gene	[39,40]
LMT	Easy operation; transient transfection; wide range of cell types	Combining other methods for cell wall treatment; only a single copy of the gene is integrated into the chromosome	Through LMT of *A. bisporus*, the *ACO* gene was verified, and the ethylene biosynthesis pathway was explored	[41,42]
EP	Easy to operate; high copy number of inserted DNA; suitable for a variety of cell types	The cells are highly damaged and inefficient; requires special equipment	The *pFTXHg* plasmid was constructed from the *ftx* gene of *F. filiformis* and was transformed by EP, and high conversion efficiency was obtained	[37,43]

ATMT: *Agrobacterium tumefaciens*-mediated transformation; PMT: protoplast-mediated transformation; LMT: liposome-mediated transformation; EP: electroporation transformation.

**Table 2:** A case study of gene function in edible and medicinal fungi using the ATMT technique.

Species	Gene	Function	Method	Vector		Refs.
				Plasmid	Promoter	
*Flammulina filiformis*	HMG-box transcription factor (*fvhom1*)	G	OE	pBHG-*fvhom1*	*A. bisporus gpd*	[44]
	Transcription factor (*Ste12-like*)	E	OE	Ste12-like^OE^	P*gpd*	[45]
	Fungal immunomodulatory proteins (*FIP-fve*)	G	OE	pCAMBIA1301-pGPD-FIP-*fve*	*pGPD*	[46]
	Fungal immunomodulatory proteins (*FIP*)	G	RNAi	pTCK303-fve (F)-fve (R)	*Ubi-1*	[47]
	Transcription factor (*FvHmg1*)	G	RNAi	Fvhmg1-RNAi	P*gpd*	[48]
	Adenosine deaminase (*Fv-ada*)	G	RNAi	pFungiway-*Fv-ada*	P*gpd*	[49]
	Heterotrimeric G protein α subunits (*FfGa1*)	E	OE RNAi	*FfGa1*-*OE* *FfGa1*-*RNAi*	P*gpd*	[50]
*Lentinus edodes*	Fungal Hsp20 protein (*hsp20*)	E	OE	pEHg-gdp-*hsp20*	*L. edodes gpd*	[51]
	Mating-type gene (*LeHD1*)	G	OE RNAi	*LeHD1-OE* *LeHD1-RNAi*	*L. edodes gpd*	[52]
	HMG-box transcription factor (*Lelcrp1*)	B	RNAi	pCAMBIA1300-g-*lelcrpl*	*L. edodes gpd*	[53]
	Heat shock protein 40 (*LeDnaJ*)	E	RNAi	pCAMBIA1300-g-*dual*	*L. edodes gpd*	[54]
	Anthranilate synthase (*TrpE*)	E	RNAi	pCAMBIA1300-g-*trpE*	*L. edodes gpd*	[55]
	Flavin-containing monooxygenases (*YUCCA*)	E	RNAi	pCAMBIA1300-g-*YUCCA8*	*L. edodes gpd*	[56]
	Tryptophan synthase (*LetrpB*)	E	RNAi	pCAMBIA1300-g-*LetrpB*	*L. edodes gpd*	[57]
*Ganoderma lucidum*	Delta 9 fatty acid desaturase (*D9desA*)	A	OE	OE::*D9desA*	*G. lucidum gpd*	[58]
	Acelyl-CoA acelyltransferase (*Gl*-*aact*)	A	OE	pGl-*aact*	*G. lucidum gpd*	[59]
	3-hydroxy-3-methylglutaryl-CoA reductase (*HMGR*)	A	OE	pJW-t*HMGR*	P*gpd*	[60]
*Antrodia* *cinnamomea*	2,3-Oxidosqualene Cyclase (*OSC*)	A	OE	pCAM-*AcOSC*	*CaMV35s*	[61]
*Pleurotus* *ostreatus*	Transcription factor (*Pofst3*)	G	OE RNAi	pPo-GPD-*Pofst3*^+^ pPo-GPD-*Pofst3*^−^	*P. ostreatus gpd*	[62]
*Cordyceps militaris*	Glutathione peroxidase (*gpxA*)	E	OE	pDHt-*gpd*-*bar*	*A. nidulans gpdA*	[63]

G: growth and development; E: environmental stress response; B: biodegradation; A: biosynthesis of functionally active substances; OE, overexpression; RNAi: RNA interference.

**Table 3:** A list of genes that regulate the development of mycelia and fruiting bodies in edible and medicinal fungi.

Species	Name	Category	Function	Refs.
*Pleurotus ostreatus*	*Vmh2, Vmh3*	Hydrophobin	Regulate the fungus to form aerial hyphae	[129]
	*polectin2*	Lectin	Promote low-temperature resistance of fruiting body	[130]
	*fst3*	Transcription factor	Regulation of primordium and fruiting body development	[131]
	*PoHMG11*	Transcription factor	Regulation of fruiting body maturation	[132]
	*PoGat1*	Transcription factor	Promote lignin decomposition and fruiting body formation	[133]
	*Pofst3*	Transcription factor	Promote the growth and development of fruiting bodies	[62]
	*Lacc2*	Laccase	Promote the growth of aerial hyphae, primordia, and fruiting bodies	[134]
	*Mnsod1*	Manganese superoxide dismutase	Promote the development of fruiting bodies and enhance mycelia tolerance to heat stress	[135]
*Flammulina filiformis*	*Hyd9*	Hydrophobin	Promote mycelia and fruiting body development	[136]
	*Fv-JRL1*	Lectin	Increase aerial hyphae and promote fruiting body development	[137]
	*Fvclp1*	Transcription factor	Involved in sexual reproduction and fruiting body development	[138]
	*ste12-like*	Transcription factor	Increase aerial hyphae and promote fruiting body development	[45]
	*pdd1*	Transcription factor	Promote fruiting body development and increase yield	[139]
	*FvHmg1*	Negative transcription factor	Negatively regulate the fruiting body development	[48]
	*LFC1*	Negative transcription factor	Negatively regulates fruiting body development and yield	[140]
	*fvopt1, fvopt2*	Oligopeptide transporter	Regulation of primordium and fruiting body development	[141]
	*Fvcpc2*	WD40 Protein	Positively regulates the development and yield of fruiting bodies	[142]
	*fv-gs6*	β-1,6-glucan synthase	Promote cell wall synthesis and stalk elongation process	[143]
	*Fvpal*	Phenylalanine Ammonia-Lyase	Involved in fruiting body growth and development	[144]
	*FfPkac*	Adenylate-dependent protein kinase A pathway	Promote mycelia and fruiting body development	[145]
	*ffccp*	Cytochrome C peroxidase	Promote elongation of fruiting body stalk	[146]
*Ganoderma lucidum*	*crzl*	Transcription factor	Involved in fruiting body growth and development	[147]
	*GlPacC*	Transcription factor	Involved in mycelium growth and fruiting body development	[148]
	*GlSwi6*	Transcription factor	Promote mycelium growth and fruiting body development	[149]
	*GlSlt2*	Mitogen-activated protein kinases	Regulates mycelium growth, fruiting body development, and cell wall integrity	[150]
*Cordyceps militaris*	*Cmhyd4*	Hydrophobin	Negative regulation of fruiting body growth and development	[151]
	*CMLec3*	Lectin	Involved in fruiting body growth and development	[152]
	*Chi1*, *Chi4*	Chitinase	Promote fruiting body growth and development	[153]
*Hypsizygus marmoreus*	*HADA-1*	Transcription factor	Promote mycelium growth and fruiting body development	[154]
	*lcc1*	Laccase	Promote fruiting body growth and development	[155]
*Coprinopsis cinerea*	*CcNsdD2*	Transcription factor	Promote secondary mycelia and fruiting body growth and development	[33]
	*Cc.Cdc3*	a homolog of *S. cerevisiae* CDC3 septin	Promote the elongation of fruiting body stalk cells	[156]
*Agaricus bisporus*	*c2h2*	Transcription factor	Shorten fruiting body development period	[157]
	*acdS*	1-aminocyclopropane-1-carboxylic acid (ACC) deaminase (AcdS)	Promote primordium initiation and fruiting body development	[158]
*Pleurotus pulmonarius*	*Ppcsl-1*	Transcription factor	Regulates fruiting body development	[159]
*Volvariella volvacea*	*Vvrin1*	Transcription factor	Promote mycelium growth and pigment accumulation, elongation of fruiting stalk, and opening of the lid	[160,161]
	*VvHox1-VvHox8*	Transcription factor	Affect the elongation of fruiting bodies and the formation of primordia	[162]
	*vvaao1*	Aryl Alcohol Oxidase	Promote the formation and development of fruiting bodies	[163]
*Lentinula edodes*	*lcc1*	Laccase	Involved in fruiting body growth and development	[164]
*Auricularia cornea*	*AcveA*	Velvet factor family protein	Regulation of fruiting body pigment synthesis	[165]
*Pholiota microspora*	*PnGcs*	α-glucosidase	Involved in fruiting body development	[166]
*Griflola frondosa*	*Rho1*	β-1,3-glucan synthase	Promote mycelium growth	[167]

**Table 4:** A list of genes related to the environmental response of edible and medicinal fungi.

Type	Species	Name	Category	Function	Refs.
Temperature	*V. volvacea*	*VvCAT1*	Catalase	Low-temperature stress defense response	[179]
	*V. volvacea*	*Mn-SOD*	Mn-superoxide dismutase	Heat, cold, and salt stress tolerance	[180]
	*L. edodes*	*LeMnP1*	Manganese peroxidase	Rapid growth recovery under high-temperature stress	[181]
	*L. edodes*	*LeDnaJ*	Heat shock protein 40	Enhance the heat resistance of mycelium	[54]
	*L. edodes*	*YUCCA8*	Flavin-containing monooxygenases	Enhance the heat resistance of mycelium	[56]
	*L. edodes*	*TrpB*	Trytophan synthase	Defense response to heat stress	[57]
	*L. edodes*	*LetrpE*	Anthranilate synthase	Enhance the heat resistance of mycelium	[55]
	*F. filiformis*	*FfGa1*	Heterotrimeric G protein α subunits	Strong tolerance to heat stress and maintenance of cell wall integrity	[50]
Light	*C. militaris*	*Cmwc-1*	Blue-light receptor	Colony pigmentation and promote conidia production	[182]
	*S. commune*	*Wc-1* *Wc-2*	Blue light complex transcription factor	Inhibit vegetative growth Phototoxic protection	[183]
	*Isaria* *cicadae*	*Icwc-1*	Blue light receptor	Regulation of fruiting body, pigment, and enzyme synthesis	[184]
	*F. filiformis*	*FfWc1 FfWc2*	Blue light and its receptor white collar complex	Regulatory morphogenesis	[185]
	*G. frondosa*	*Gf.BMR1*	Transcription factor	Fruiting body development and pigment accumulation	[186]
	*L. edodes*	*abl-D*	Abnormal browning related to light	Brown film formation of mycelial tissue	[187]
	*A. bisporus*	*Pabs*	Para-aminobenzoic acid synthase	Enhance UV tolerance	[188]
Metal stress	*M. esculenta*	*ATX1*	Copper chaperones	Regulation of cadmium metabolism	[189]
	*L. edodes*	*LeAmy*	α amylase	Enhance cadmium tolerance	[190]

**Table 5:** Genes affecting the biosynthesis of ganoderma acids.

Gene	Mode of Action and Change of GA Content	Refs.
Alternative oxidase (*AOX*)	Silent *AOX*, ROS content ↑, GA content ↑	[206]
Transcription factor (*GCN4*)	Silent *GCN4*, ROS content ↑, GA content ↑	[207]
AMP-activated protein kinase homolog (*GlSnf1*)	Silent *GlSnf1*, ROS content ↑, GA content ↑	[208]
Telomerase reverse transcriptase (*GlTert*)	Silent *GlTert*, ROS content ↑, GA content ↓	[209]
Calcium-permeable channel (*Cch*)	Silent *Cch*, Ca^2+^ content ↓, GA content ↓	[210]
Nitrate reductase (*NR*)	Silent NR, GA content ↑	[211]
Glutamine synthetase (*GS*)	Silent *GS*, GA content ↓	[212]

Note: ↑: increase; ↓: decrease.

## Data Availability

Data are contained within the article.
